# Predictors of Re-Engagement after Relapse in a Tobacco Quit Line Intervention: Secondary Analysis from a Randomized Clinical Trial

**DOI:** 10.3390/ijerph20021229

**Published:** 2023-01-10

**Authors:** Kara P. Wiseman, Chase A. Aycock, Indika Mallawaarachchi, Xin-Qun Wang, Daniel G. Cassidy, Marc A. Patience, Melissa A. Little, G. Wayne Talcott, Robert C. Klesges

**Affiliations:** 1 Department of Public Health Sciences, School of Medicine, University of Virginia, Charlottesville, VA 22908, USA; 2 Wilford Hall Ambulatory Surgical Center, Clinical Health Psychology, San Antonio, TX 78236, USA; 3 Malcolm Grow Medical Clinics and Surgery Center, Clinical Health Psychology, Prince George’s County, MD 20762, USA

**Keywords:** smoking cessation, quit line, re-engagement, mHealth

## Abstract

People who smoke often make several quit attempts before successfully maintaining abstinence. Therefore, incorporating re-engagement for people who fail to initially quit could increase quit attempts and ultimately increase cessation rates. Within the context of quit line-based interventions, it remains unknown what characteristics are associated with re-engagement. The purpose of this study was to assess associations between demographic and motivational characteristics, tobacco use, and initial intervention engagement with re-engagement in a tobacco quit line intervention. Among 372 adults who reported smoking three months after initiating a quit line-facilitated quit attempt as part of a larger randomized clinical trial, associations between personal characteristics (e.g., age, gender, nicotine dependence, and confidence in their ability to quit smoking) and initial intervention engagement (number of completed counseling sessions and use of nicotine replacement therapy (NRT)) with re-engagement (accepting an offer to re-initiate the quit line intervention) were determined using multivariable logistic regression modeling. Compared to non-White participants, White participants had lower odds of re-engaging (OR: 0.42, 95% CI: 0.23, 0.75). Number of initial counseling sessions completed was associated with re-engaging. NRT use during the initial intervention was not associated with re-engaging. Initial intervention engagement is important in the process of re-engagement, specifically attending counseling sessions. Exploration of associations between initial intervention engagement and potentially modifiable motivational factors is needed to be potentially leveraged in future interventions to maintain continued engagement in cessation among adults who smoke.

## 1. Introduction

Cigarette smoking remains the leading cause of preventable death in the US [1]. An estimated 12.5% of US adults report current smoking [2], but most recently available evidence suggests that a full 68% are interested in quitting [3]. Quit line cessation interventions are effective and have the potential to reach people who smoke across the country. However, a single use of a quit line intervention is often insufficient for many, as people who smoke must often make multiple attempts before successfully quitting [4,5]. Fortunately, more than 60% of people who smoke report interest in re-engagement with smoking cessation after failing to quit [6,7]. Given the widespread need for multiple quit attempts and reported interest in continued support, incorporating re-engagement for those who fail to initially quit could increase quit line-facilitated quit attempts and ultimately increase cessation rates. Further, incorporating elements of tailoring or personalization could enhance successful re-engagement and quit line performance.

To inform tailoring strategies, the characteristics of people who smoke who are interested in re-engaging following a failed quit attempt need to be identified. Insight concerning who is likely to re-engage may enable resource-limited quit lines to target outreach strategies for maximum re-engagement. To our knowledge, two previous studies have examined predictors of re-engagement in a tobacco quit line. Using data from the Arizona Quit line, Nair et al. found that individuals who chose to re-engage had higher odds of both mental health and chronic health diagnoses [8]. They also found that male gender, referral to the quit line by a health care provider, cohabitation with other smokers, and possession of medical insurance were inversely associated with re-engagement. Beebe et al. considered the association between initial program selection and re-engagement in the Oklahoma Quit line [9]. People using the quit line had the option to receive nicotine replacement therapy (NRT) and were asked to enroll in either a more intensive multi-call telephone intervention or enroll in less intensive cessation services (less-intensive options included a text-messaging program, an email program, and/or a booklet). Individuals who selected to enroll in a less-intensive cessation service were more likely to re-engage compared to those who initially selected the multi-call telephone intervention. When comparing characteristics between those who re-engaged and those who did not, univariate analyses revealed that a higher proportion of those re-engaged were older, more likely to be men, had an income less than USD 35,000, and had initially enrolled via telephone call (vs. the web). When examining predictors of re-engagement among the subset of callers to the quit line who had selected to initially use less-intensive cessation services and who had also used NRT during their quit attempt, the strongest predictor of re-engagement was receiving an NRT counseling phone call.

Thus, the previous literature has assessed associations between demographic characteristics, tobacco use characteristics, and the initial mode of intervention with re-engagement. However, the paucity of research in this area warrants continued evaluation. Further, there remain additional areas for investigation that have not been previously considered. Specifically, it is unclear if engagement in the initial intervention, or interactions between use of initial intervention components (e.g., attending counseling sessions and using NRT) influence re-engagement. It is also unknown if confidence in quitting or reasons for quitting influence re-engagement. Consequently, this study sought to assess the association between demographic and motivational characteristics, tobacco use, and initial intervention engagement with re-engagement in a tobacco quit line intervention.

## 2. Materials and Methods

### 2.1. Data and Study Population

This is a secondary data analysis from a large clinical trial of 612 adults who smoke designed to determine the efficacy of three re-engagement strategies on long-term (12 months) smoking cessation [10]. Briefly, TRICARE beneficiaries (i.e., active military personnel, retirees, and their dependents) were recruited for a quit line intervention via printed materials posted at US Air Force bases and electronic media. All participants completed a telephone baseline survey and then received a 4 week proactive quit line intervention in the first-phase treatment, which included eight weeks of mailed nicotine replacement therapy (NRT). Follow-up assessments were completed by telephone at 3- and 12-months after study enrollment. At the time of the 3-month assessment, any participants who reported current smoking (e.g., failed to initially quit or had relapsed to smoking) were offered the opportunity to re-engage in cessation services. Those who chose to re-engage were randomized into one of three arms for the second-phase treatment: (1) repeat the initial intervention, (2) a step-down (rate reduction) intervention, or (3) their choice of repeating the initial intervention or trying the step-down intervention. The trial was approved by the US Air Force Wilford Hall Medical Center IRB and registered on clinicatrials.gov (NCT02201810). For these analyses, we excluded all participants who reported smoking abstinence at three months (*n* = 226) or who were lost to follow-up before the three-month follow-up (*n* = 14), leaving *n* = 372 in the analytic sample. Data for the current study included information captured during the baseline and 3-month assessments. 

### 2.2. Measures

The primary outcome of interest was the participant’s decision to re-engage at three months (yes vs. no) based on responses to the three-month follow-up assessment, thereby making another quit line-facilitated quit attempt, or to decline the opportunity to re-engage, including overt decline to re-engage (*n* = 130), and passive refusal due to non-response (despite more than eight attempts to contact the participant by intervention staff) to the three-month follow-up assessment (*n* = 108).

Several independent variables of interest were included, which were all measured at the baseline assessment. Nicotine dependence was assessed using the Fagerström Test for Nicotine Dependence [11], and calculated as total score per participant. Confidence in quitting was assessed by asking participants, “How confident are you that you will quit smoking some day?” Response options were a five-point Likert-type scale dichotomized to extremely confident vs. all other responses [12]. Reasons to quit smoking were assessed by providing participants with nine items (e.g., quitting to save money, quitting due to pressure from others, and quitting so that hair and clothes won’t smell) and then asking, “People have different reasons for wanting to quit smoking. On a scale of 1 to 5 with 1 being Not at all True and 5 being Extremely True, please indicate how true each of the following is for you.” [13]. Responses to the five-point Likert-type scale were recoded as three-level or dichotomized variables per the distribution of each item. The number of counseling sessions participants completed (0, 1, 2, 3, or 4) and if the participant made use of NRT during the initial intervention (yes or no) were used to operationalize engagement in the initial intervention. Lastly, demographic characteristics (i.e., age, gender, race, education, marital status, and military status) were considered.

### 2.3. Statistical Analyses

All analyses were performed using SAS 9.4 (SAS, Cary, NC, USA). Continuous variables were summarized using median and interquartile range and compared between participants who re-engaged vs. did not re-engage a using Wilcoxon rank sum test. Categorical variables were summarized using frequency and percentages and compared by re-engagement status using Fisher’s exact test. A multivariable logistic regression model to determine correlates of re-engagement (outcome referent = did not re-engage) was created, which included any independent variable of interest found to be associated with re-engagement in the univariate statistics using a cut-off of *p*-value of ≤0.20 [14]. To determine the impact of initial intervention engagement with re-engagement, an interaction between counseling sessions completed and NRT use was also tested; however, there was no statistical interaction effect (*p* = 0.980). Therefore, the interaction term was not included in the final model. The C-index of final model was 0.76, indicating that the multivariable logistic regression model had good predictive discrimination power to model subjects who re-engaged vs. did not re-engage [15].

## 3. Results

Among participants who did not re-engage, the majority were male (56.7%), White (83.1%), married or partnered (65.6%), and active duty or retired military (63.9%, Table 1). A higher percentage of participants who re-engaged used NRT during the initial intervention (70.2% vs. 46.2% for re-engaged vs. not, respectively, *p* < 0.001) and completed all four initial counseling sessions (73.0% vs. 39.6% for re-engaged vs. not, respectively, *p* < 0.001). A higher percentage of participants who re-engaged reported greater confidence in quitting at baseline (57.9% vs. 44.7% for re-engaged vs. not, respectively, *p* = 0.017, Table 2). There were several differences between those who did, and did not, re-engage with respect to reasons for quitting. For example, a higher percentage of participants who re-engaged reported quitting to be a good role model for others (76.1% vs. 64.6% for re-engaged vs. not, respectively, *p* = 0.027).

Primary Results: Multivariable results indicated that race, baseline confidence in quitting, and initial counseling sessions completed were associated with re-engagement (Table 3). Specifically, compared to non-White participants, White participants had lower odds of re-engaging (OR: 0.42, 95% CI: 0.23, 0.75). Participants who had the highest levels of confidence at baseline had 1.84 the odds of re-engaging compared to participants with lower levels of baseline confidence in quitting (95% CI: 1.12, 3.03). Participants who did not complete all the initial counseling sessions had lower odds of re-engaging at three months. NRT use during the initial intervention was not associated with re-engagement (OR: 1.55, 95% CI: 0.80, 3.02). Specific reasons to quit smoking were not associated with re-engagement in the final model. 

## 4. Discussion

The purpose of this study was to identify interpersonal and intervention use characteristics associated with re-engagement among adults participating in a quit line intervention study. To our knowledge, this study is the first to consider personal, tobacco use, motivation, and initial intervention utilization factors that may be associated with re-engagement in a quit line intervention. We found that, among all possible characteristics of interest, non-White race and high levels of baseline confidence in quitting were associated with re-engagement at three months. Engagement with the initial intervention was also associated with re-engagement, specifically the number of initial counseling sessions completed. These results provide new information about why some adults who smoke who fail to quit may ultimately choose to re-engage in a quit line intervention, establishing new potential directions for future studies.

Some studies have attempted to increase re-engagement in quit line services among adults who smoke who initially fail to quit. For example, Vickerman et al. randomized callers to the Minnesota Quit Line into receiving or not receiving re-engagement outreach [16]. Outreach was conducted using multiple methods (i.e., phone, email, and/or text message) at one, two, or three months after initial engagement. Proactively re-engaging people who had failed to quit resulted in five-fold greater odds of re-engagement than those randomized to reactive (i.e., participant-initiated) contact [16]. In a series of studies by Carlini et al., using proactive re-engagement through Interactive Voice Response technology, resulted in participants having a greater odds of re-engagement relative to an automated screening of current tobacco use [17,18,19]. However, our results provide information that could be used when targeting re-engagement strategies to specific groups, which might be necessary to further increase re-engagement. In our study sample, White participants were less likely to re-engage than non-White participants. However, none of the previous research has identified race as being associated with re-engagement. Similarly, while Nair et al. found that men were more likely to re-engage [8], neither our study nor Beebe et al. found an association between gender and re-engagement [9]. With this inconsistent evidence, it is unclear if targeting demographic characteristics would be an effective way to increase re-engagement within quit line interventions. We are aware of only one study that has attempted to increase re-engagement by focusing on specific demographic groups. Carlini et al. compared two mailed interventions, one was tailored to the participant’s race/ethnicity and one the other was generic [19]. However, there was no difference in re-engagement between generic and tailored re-engagement messages. It is worth noting though that the rate of re-engagement with the mailed intervention was low to begin with (0.53–0.67%); thus, it is possible that targeted interventions using a different format (e.g., phone calls or text messages) may yield different results. 

We also identified previously unconsidered motivational factors that were associated with re-engagement at three months. Specifically, participants with higher levels of baseline confidence in quitting were more likely to re-engage at three months, which was roughly 6 weeks after the initial intervention ended. It is noteworthy that baseline confidence would produce such an effect, especially within a sample of adults who were all unsuccessful in their first quit attempt. It is possible that increasing confidence in quitting by providing motivational messages by counselors early in quit line counseling may increase their engagement in the initial intervention as well as the likelihood that those still smoking after their initial quit attempt will subsequently be open to re-engagement. Tangentially, Danaher et al. found that positive changes in ratings on a five-point Likert scale of one’s confidence of being tobacco-free in 1 year mediated the relationship between a web-based tobacco intervention and abstinence at 3- and 6-month follow-up [20]. Relatedly, it is possible that motivational interviewing at the time of a proactive re-engagement call for callers who initially report lower confidence in quitting, might increase their self-efficacy in quitting and their subsequent desire to re-engage [20,21].

Our finding that participants who completed more initial intervention quit line counseling sessions had increased odds of re-engagement with the quit line intervention suggests that increasing initial intervention engagement could be a promising strategy to maximize re-engagement following an unsuccessful quit attempt. To our knowledge, no studies have sought to bolster initial engagement of quit line callers with the goal of improving chances of eventual re-engagement intervention. Beebe et al. (2020) found that selection of a less intensive option by callers for their initial intervention predicted increased likelihood of re-engagement [9]. It could be that having a positive experience with the initial intervention led to increased interest in re-engaging in services even after failing to quit. In the context of our quit line intervention, it is possible that individuals who engaged more in the initial intervention may have grown more familiar with the rationale of the treatment, increasing their awareness of the relevance and potential utility of continuing treatment even after failing to quit. Future studies should continue to investigate the meaning of cessation intervention engagement across types of interventions and consider what constructs may influence engagement with counselor-based cessation interventions. 

This study estimated the effect of initial intervention engagement on re-engagement at three months independent of baseline motivational factors such as confidence in quitting some day and specific reasons for quitting smoking, including quitting to be a good role model and quitting to control one’s life. Nevertheless, there remains a potential that motivation has an influence on both initial intervention engagement and interest in re-engagement after relapse. Due to missing data at three months, we were not able to consider motivational items at the time of re-engagement. However, descriptively at three months, a higher percentage of participants who chose to re-engage reported seeing extreme benefit in quitting for their health (65.6% vs. 49.3% for re-engaged and non-re-engaged, respectively, *p* = 0.026) and were extremely confidence in quitting someday (91.4% vs. 81.7% for re-engaged and non-re-engaged, respectively, *p* = 0.067) It could also be informative to explore patterns of motivation assessed during the quitline intervention to further explore trajectories in motivation and its association with subsequent re-engagement. Lastly, those in the re-engaged group had higher mean levels of reporting stress as a reason for relapse (mean 4.00 and 3.61 for re-engaged and non-re-engaged, respectively, *p* = 0.014), which suggests that those who re-engaged may have directly seen the benefit of having a trained counselor to guide them through the quitting process. Future research should consider exploration of these constructs in a fully powered manner to determine their relevance in the decision to re-engage when adjusting for other characteristics.

This study has several limitations that need to be considered. First, these results are from a secondary analysis of data from a randomized trial [10], missing data precluded the inclusion of several potentially relevant constructs and, due to small cell sample sizes, we were unable to disaggregate across other races. Future research with more diverse populations is needed to consider this association using more nuanced comparisons. Additionally, we were unable to plan for appropriate statistical power; as a result, our models may be underpowered to detect associations with re-engagement. We were, nevertheless, still able to address the study aims and have identified several new avenues for exploration to potentially increase re-engagement-focused quit line-facilitated cessation studies. Additionally, the results of this study may be of limited generalizability, as the study population consisted of active duty service members, retired military personnel, and military family members. It will be important to explore the results described here in samples comprising non-military populations to determine the replicability of our findings across contexts. In this last respect, while our sample was predominantly active duty and retired military, the inclusion of military family members allowed us to examine the association between military history and re-engagement. A lack of relationship between military status and re-engagement tentatively suggests that our results may, in fact, generalize to other populations of adult smokers, despite consisting of a special population.

## 5. Conclusions

This study identified predictors of re-engagement among adults enrolled in a cessation intervention who relapsed or failed to initially quit by three months after enrolling in a cessation quit line intervention study. We identified initial intervention use and personal characteristics associated with re-engagement, including non-White race and confidence in one’s ability to quit smoking someday. Our results point to the importance of initial quit line intervention engagement in the process of increasing the likelihood of re-engagement, specifically for participants who attended all offered counseling sessions. Future studies should further explore the associations between initial intervention engagement and potentially modifiable motivational factors that could be leveraged in future interventions to maintain continued engagement in the cessation process.

## Figures and Tables

**Table 1:** Demographic, tobacco use, and initial intervention use overall and by three-month re-engagement.

Characteristic	Overall (*n* = 372)	Not Re-Engaged (*n* = 238)	Re-Engaged (*n* = 134)	*p*-Value
	n (%)	n (%)	n (%)	
Age: Median (interquartile range)	49.3 (32.1, 62.0)	47.5 (31.4, 61.2)	52.6 (34.0, 63.7)	0.138
Gender				0.234
Male	202 (54.30)	135 (56.72)	67 (50.00)	
Female	170 (45.70)	103 (43.28)	67 (50.00)	
Race ^a^				0.023
Other races ^b^	76 (21.02)	40 (16.95)	36 (27.07)	
White	293 (78.98)	196 (83.05)	97 (72.93)	
Marital status				0.249
Married/Living as married	252 (67.74)	156 (65.55)	96 (71.64)	
Not together	120 (32.26)	82 (34.45)	38 (28.36)	
Military status ^a^				0.069
Dependent	145 (39.08)	86(36.13)	59(44.36)	
Active	107 (28.84)	78(32.77)	29(21.8)	
Retired	119 (32.08)	74(31.09)	45(33.83)	
Education ^a^				0.665
High school diploma or GED	87 (23.58)	59 (25.00)	28 (21.05)	
Some college/vocational school/Associates degree	192 (52.03)	119 (50.42)	73 (54.89)	
Bachelor’s degree or post college	90 (24.39)	58 (24.58)	32 (24.06)	
Fagerstrom nicotine dependence score: Median (interquartile range)	5.0 (3.0, 6.0)	5.0 (3.0, 6.0)	5.0 (3.0, 6.0)	0.338
NRT use during intervention				<0.001
No	168 (45.16)	128 (53.78)	40 (29.85)	
Yes	204 (54.84)	110 (46.22)	94 (70.15)	
Number of initial counseling sessions completed				<0.001
0	44 (11.83)	36 (15.13)	8 (5.97)	
1	49 (13.17)	38 (18.81)	11 (8.73)	
2	53 (14.25)	41 (20.30)	12 (9.52)	
3	54 (14.52)	43 (21.29)	11 (8.73)	
4	172 (46.24)	80 (39.60)	92 (73.02)	

Note: *p*-value for categorical and continuous comparisons were derived from univariate Fisher’s exact test and Wilcoxon rank sum tests, respectively. ^a^ Sum does not add to total due to missing data. ^b^ Due to small sample sizes across each race, we were unable to differentiate between non-White categories. Therefore, the “Other races“ category includes Black, Asian, American Indian/Alaskan native, Pacific Islander, Multiple races, and those who reported other or preferred not to disclose.

**Table 2:** Baseline confidence in quitting and reasons for quitting by three-month re-engagement.

Characteristic	Not Re-Engaged (*n* = 238)	Re-Engaged (*n* = 134)	*p*-Value
	n (%)	n (%)	
How confident are you that you will quit smoking some day? ^a^			0.017
Not extremely confident ^b^	131 (55.27)	56 (42.11)	
Extremely confident ^c^	106 (44.73)	77 (57.89)	
Quit smoking to save money			0.900
Not true ^d^	49 (20.59)	27 (20.15)	
Neutral ^e^	36 (15.13)	18 (13.43)	
True ^f^	153 (64.29)	89 (66.42)	
Quit smoking because I am getting pressure from others			0.838
Not true ^d^	130 (54.62)	69 (51.49)	
Neutral ^e^	44 (18.49)	26 (19.40)	
True ^f^	64 (26.89)	39 (29.10)	
Quit smoking so that my hair and clothes won’t smell			0.177
Not true ^d^	73 (30.67)	38 (28.36)	
Neutral ^e^	59 (24.79)	24 (17.91)	
True ^f^	106 (44.54)	72 (53.73)	
Quit smoking because it is too difficult to find a place to smoke			0.785
Not true ^d^	182 (76.47)	104 (77.61)	
Neutral ^e^	33 (13.87)	20 (14.93)	
True ^f^	23 (9.66)	10 (7.46)	
Quit smoking to improve my overall health			1.000
Not very true ^g^	18 (7.56)	10 (7.46)	
Very true ^h^	220 (92.44)	124 (92.54)	
Quit smoking to be a good role model for others ^a^			0.027
Not true or neutral ^i^	84 (35.44)	32 (23.88)	
True ^f^	153 (64.56)	102 (76.12)	
Quit smoking so I can be in control of my life			0.039
Not true or neutral ^i^	62 (26.05)	22 (16.42)	
True ^f^	176 (73.95)	112 (83.58)	
Quit smoking to improve my overall physical fitness			0.487
Not true or neutral ^i^	18 (7.56)	6 (4.48)	
Somewhat true ^j^	29 (12.18)	15 (11.19)	
Very true ^h^	191 (80.25)	113 (84.33)	
Quit smoking because smoking may have a negative effect on my career ^a^			1.000
Not true or neutral ^i^	180 (75.63)	101 (75.94)	
True ^f^	58 (24.37)	32 (24.06)	

Note: *p*-value derived from univariate Fisher’s exact test. Responses to the five-point Likert-type scale were recoded as three-level or dichotomized variables per the distribution of each item. ^a^ Sum does not add to total due to missing. ^b^ Not extremely confident = response of 1–4. ^c^ Extremely confident = response of 5. ^d^ Not true = response of 1 or 2. ^e^ Neural = response of 3. ^f^ True = response of 4 or 5. ^g^ Not very true = response of 1–4. ^h^ Very true = response of 5. ^i^ Not true or neutral = response of 1–3. ^j^ Somewhat true = response of 4.

**Table 3:** Association between personal characteristics, and initial intervention engagement with re-engagement at three months.

Characteristic	OR (95% CI)
Age (a unit increase)	1.01 (0.99, 1.03)
Male (vs. Female)	0.97 (0.44, 2.12)
White (vs. Other races)	0.42 (0.23, 0.75)
Education	
College Degree (vs. High school diploma or GED)	1.02 (0.50, 2.08)
Some college (vs. High school diploma or GED)	1.16 (0.62, 2.15)
Military status	
Dependent (vs. Active)	1.35 (0.52, 3.50)
Retired (vs. Active)	1.25 (0.54, 2.85)
Married or partnered (vs. not)	1.28 (0.75, 2.21)
Total Fagerstrom score (a unit increase)	1.09 (0.97, 1.21)
Extremely confident in quitting some day (vs. not)	1.84 (1.12, 3.03)
Quit smoking to be a good role model for others (vs. not)	1.71 (0.95, 3.08)
Quit smoking so I can be in control of my life (vs. not)	1.23 (0.65, 2.34)
Quit smoking so that my hair and clothes won’t smell (vs. not)	1.08 (0.65, 1.80)
Counseling sessions completed	
0 (vs. 4)	0.21 (0.07, 0.60)
1 (vs. 4)	0.38 (0.15, 0.97)
2 (vs. 4)	0.29 (0.13, 0.65)
3 (vs. 4)	0.19 (0.09, 0.43)
Used NRT during initial intervention (vs. no)	1.55 (0.80, 3.02)

OR: odds ratio; CI: confidence interval. Due to small sample sizes across each race, we were unable to differentiate between non-White categories. Therefore, the “Other races“ category includes Black, Asian, American Indian/Alaskan native, Pacific Islander, Multiple races, and those who reported other or preferred not to disclose.

## Data Availability

This study was pre-registered at clinicaltrials.gov: NCT02201810. The analysis plan was not formally pre-registered. De-identified data from this study are not available in a public archive. De-identified data from this study will be made available (as allowable according to institutional IRB standards) by emailing the corresponding author. Analytic code used to conduct the analyses presented in this study are not available in a public archive. They may be available by emailing the corresponding author. Materials used to conduct this study are not publicly available.
